# Psychological Impact of the COVID-19 Pandemic on Students, Assistants, and Faculty of a Dental Institute of Saudi Arabia

**DOI:** 10.3390/ijerph182413366

**Published:** 2021-12-19

**Authors:** Saqib Ali, Saman Tauqir, Faraz Ahmed Farooqi, Badr Al-Jandan, Hawra Al-Janobi, Sami Alshehry, Adel Ibrahim Abdelhady, Imran Farooq

**Affiliations:** 1 Department of Biomedical Dental Sciences, College of Dentistry, Imam Abdulrahman Bin Faisal University, Dammam 31441, Saudi Arabia; drsaqiibali@gmail.com (S.A.); baljandan@iau.edu.sa (B.A.-J.); haaljanobi@iau.edu.sa (H.A.-J.); smalshehri@iau.edu.sa (S.A.); aiabdulhady@iau.edu.sa (A.I.A.); 2 Department of Physiology, Institute of Basic Medical Sciences, Khyber Medical University, Peshawar 25120, Pakistan; samantauqir@gmail.com; 3 Department of Dental Education, College of Dentistry, Imam Abdulrahman Bin Faisal University, Dammam 31441, Saudi Arabia; fafarooqi@iau.edu.sa; 4 Faculty of Dentistry, University of Toronto, Toronto, ON M5G 1G6, Canada

**Keywords:** COVID-19, stress, psychology, dentistry

## Abstract

This study aimed to assess the perceived stress levels in students, assistants, and faculty members of the College of Dentistry, Imam Abdulrahman Bin Faisal, University (IAU), Kingdom of Saudi Arabia (KSA) during the novel coronavirus disease 2019 (COVID-19) pandemic. Using the Cohen’s perceived stress scale (PSS) questionnaire (consisting of 14 items, hence called PSS-14), an online observational survey was conducted. The PSS 14 was rated on a 5-point Likert scale ranging from 0 (never) to 4 (very often). The scores ranging from 0–18 represented low stress, 19–37 represented moderate stress, and 38–56 represented high stress. The second-and third-year students were designated as junior year students, while fourth-year onwards were considered senior year students. Out of total 265 participants, 65% (173) were female, and the majority of the participants were dental students 70% (185) with a mean age of 26.71 ± 9.26 years. In the present study, the average PSS score for the participants was computed as 29.89 (range score: 0–56) which shows moderate stress levels among the respondents. The PSS score for the students was 31.03; for the faculty, it was 28, while for the assistants, it was 27.05. Among the three participant groups, the students were found more on the severe stress side (19%) (*p*-value = 0.002), and among them, the senior year students (6th year) showed significantly higher stress levels compared to the junior year students (*p*-value = 0.005). Age-wise, the participants below 20 years were most stressed (21%), followed by those 20–30 years old (18%). Female participants were more severely stressed than males (17% vs. 10%, respectively). It was concluded that the students experienced more stress, followed by the faculty members and dental assistants. In addition, younger participants, females, and senior year students were more stressed than their counterparts. Future studies directed at evaluating stress levels of these groups from different dental institutes could provide an opportunity for policymakers to offer various resources to improve their mental health.

## 1. Introduction

The coronavirus disease 2019 (COVID-19) originated as a cluster of inexact pneumonia cases in December 2019 in Wuhan, China [1]. At the present moment, it has become a global pandemic, affecting every nation of the world [2]. Experts have reported that when the COVID-19 virus infects someone, the lesions are not limited to their lungs: the virus causes viremia upon entering the human body, resulting in diverse clinical manifestations including fever, fatigue, diarrhea, and some other nonspecific signs and symptoms [3,4,5]. The COVID-19 is a highly transmissible disease [3]. Due to a high transmissibility rate, the Saudi government had to enforce social distancing at a population and individual level [6]. To prevent the rapid transmission of the disease, different measures were introduced around the country including the closure of the educational institutes, avoidance of open gatherings, and nationwide lockdown [6,7]. Due to these sudden closures, educational and professional activities were affected during the COVID-19 pandemic [8]. In fact, globally there are more than 100 countries that have reported suspension of teaching activities during the pandemic [9]. Owing to the severity of the situation, many universities halted campus-based teaching and continued with the online teaching [8]. Unfortunately, stakeholders of the institutions (students and employees) were not ready for this sudden switch, and this led to an increase in their stress levels. During the time of this public health emergency, medical caretakers, doctors, paramedics, nurses and medical students were also exposed to high levels of stress both physically and psychologically causing mental health problems [10,11]. The fear of catching the virus has aggravated psychological pressure and mental illness in the said population, making them vulnerable to high stress [12,13]. The pandemic has caused a “mental health catastrophe” causing psychiatric disorders after the COVID-19 outbreak [14]. All the communities became vulnerable and felt threatened by potential health emergencies [15], and during the time of social distancing, homeschooling, home quarantine, and work closures, people need support [16]. Quarantine has a wide range of psychological impacts on an individual’s mind, and its effects are long-lasting [16]. Previously, during the Middle East Respiratory Syndrome (MERS) outbreak, there were high levels of stress seen in the medical students of KSA [17]. Similarly, during the COVID pandemic, perceived stress among school and university students recorded in virtual classrooms was high to moderate [18]. In another study conducted on dental students in Romania, the impact of COIVD-19 was investigated, and findings demonstrated their emotional state being adversely affected [19]. Previously, health care students from the central region of KSA also reported fear and anxiety due to COVID-19 [20]. Depression, anxiety, and fear were reported in a study that was conducted on dental interns in Riyadh, KSA [21]. Considering the importance of mental health, this subject should be investigated further at dental institutions.

Dental schools cater preclinical and clinical students who attend lectures, laboratory sessions, and clinics (treating patients in their senior years). The dental faculty teach and train students both non-clinically and clinically over the period of their course and they all are assisted by dental assistants/ nurses in laboratories and clinics. The authors believe that dental schools are unique in a way that students, faculty, and dental assistants work as a team to learn, train, and treat patients in their clinical practice. Due to the involvement of students, faculty, and assistants with the patients, the fear of contracting COVID-19 is always present, and this issue needs further exploration. COVID-19 caused fear, anxiety, and stress among the academic community specifically those associated with health care [22]. Currently, there are no significant studies on psychological stress levels of dental students, assistants, and faculty after the lockdown and resumption of on-campus educational and clinical activities have begun.

Therefore, it is important to study the effects of such rapidly spreading infectious diseases on the psychological well-being of the current and future frontline warriors. Thus, the goal of the present study was to assess the perceived stress brought by the COVID-19 pandemic amongst students (undergraduates and interns), dental assistants, and the faculty members of the College of Dentistry (COD), Imam Abdulrahman Bin Faisal University (IAU), Dammam, Kingdom of Saudi Arabia (KSA). The findings of this study could help establish measures to improve the psychological well-being, and help identify the most vulnerable group so psychological intervention can be directed towards them. Additionally, the results from this study can be taken as a pathfinder to explore psychological stress among dental schools around the country for the development of effective screening tools and strategies for intervention to revitalize psychological resilience among the current and future frontline warriors.

## 2. Materials and Methods

**Figure 1:**
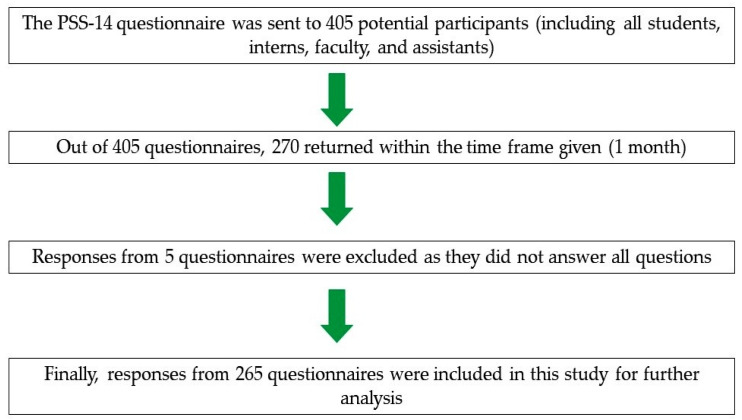
Flow chart showing the participant recruitment process of our study.

The ethics board of the college approved the study (Ref: EA-202155). The research was carried in accordance with the Helsinki Declaration. A cross-sectional web-based observational study was designed and carried out at COD, IAU, KSA from 1 to 31 March 2021. A questionnaire was uploaded online using the website QuestionPro. A consent form was attached with the survey, and confidentiality of the respondent’s information was assured. The questionnaire link was shared with the class representatives of various batches of students and with all the faculty and assistants via Email, WhatsApp^TM^, Facebook^TM^, and other social media websites, and they were encouraged to share it with their colleagues. Thus, the link was shared through all the primary sources of communication to reach many subjects. The participant recruitment process adapted in our study is shown in Figure 1.

Upon clicking the link, the participants were directed to the consent section of the study. After they agreed to the survey, they first filled in the demographic details, which included age, gender, educational level, and residence details (living with family or in a dorm, optional question). After filling in these details, a set of questions appeared in a sequence which the participants had to answer. In the current study, we utilized an Arabic version [23] of the Cohen’s perceived stress scale (PSS 14) [24] to assess our participants’ stress responses during the COVID-19 pandemic. This stress scale was used during the COVID-19 pandemic and it was shown to be effective in assessing the stress levels of the participants [25,26]. The PSS 14 was rated on a 5-point Likert scale ranging from (0 = never) to (4 = very often). Seven positive items were reverse coded (e.g., 0 = 4, 1 = 3, 2 = 2, etc.), which included items 4, 5, 6, 7, 9, 10, and 13, described as positively stated items in the questionnaire. The total PSS score was obtained by summing all 14 items’ scores, and a higher total score indicated higher perceived stress. The scores ranging from 0–18 were considered as low stress, 19–37 were considered as moderate score, and the scores ranging from 38–56 were considered as high stress, as coded earlier by Higgins [27]. The age range was divided into a group of four ranging between 18–20, 21–25, 26–30, and 31 or above. Second- and third-year students were designated as junior year students, while those of fourth-, fifth-, and sixth year, and interns, were considered as senior year students. The inclusion criteria were that the dental students (undergraduate students and interns), faculty, and dental assistants must be studying or working in our university, and the participants voluntarily responded to the survey.

### Statistical Analysis

Data were exported to Excel from Google Docs initially and were then transferred to SPSS (version 22, IBM, Chicago, USA) for analysis. Sociodemographic characteristics of the participants were presented in the form of frequencies, percentages, mean, and standard deviation (where appropriate). A chi-square test was performed between demographics and perceived stress categories (low, moderate, and high) to compare them. Mean PSS score was compared for age categories, participants group, and level of education using One Way ANOVA. Logistic regression models were created to evaluate the crude association between PSS Score (dependent) and demographical characteristics (independent variables). Predictors with less than <0.10 were retained for the final regression model. All individual predictors were combined, and an unstandardized B coefficient, 95% CI, was presented. *p*-values ≤ 0.05 were considered significant.

## 3. Results

Our study had a response rate of 65%. In our study, more than half of the participants were female (65%), and majority of the participants (70%) were dental students with a mean age of 26.71 years. Among the dental students, 19% were junior students (second year), followed by fourth-year students and interns (18% each). Faculty participants were found to be the lowest among the respondents (15%) (Table 1).

The majority of participants showed moderate stress, and they were aged >40 years. Participants below 20 years were most stressed (21%), followed by 20–30 years old (18%), and the eldest participant group of the study showed no severe stress levels (0%). Age categories were significantly associated with the level of stress (*p*-value = 0.043). Female participants were more severely stressed than males (17% vs. 10%, respectively), and the association between gender and level of stress was also statistically significant (*p*-value = 0.040). Similarly, among the participants’ group, the students were found more on the severe stress side (19%) (*p*-value = 0.002), and among them, the senior year level (6th year) showed significantly higher stress level compared to junior year students (*p*-value = 0.005) (Table 2).

In the present study, the average PSS score for the participants was computed as 29.89 (range score: 0–52) which explains the moderate stress level seen in the participants. All the participants’ groups when evaluated by age and academic year levels, showed a significant mean difference in PSS score (Table 3). The average PSS score significantly reduced with the increase in age (*p*-value = 0.001). Stress score was significantly higher among the students as compared to the faculty (31 vs. 28, *p*-value = 0.001). Among the students, the highest PSS score (34.41) was recorded among the most senior students (6th year) whereas, the lowest score (30) was recorded among the most junior students (2nd year), and the differences were statistically significant (*p*-value = 0.001).

Hosmer and Lameshow test statistics support the model fitness (Ҳ^2^ = 6.003, *p*-0.199), and small Negelkerke R-square values support the good of fit test (R^2^ = 0.091). Logistic regression revealed that female students were more likely to have high stress compared to the male participants (OR: 4.89, *p*-value = 0.027), whereas the increased-age participants were less likely to have stress compared to the younger age group participants (less than 20 years old) (Table 4).

## 4. Discussion

In the present study, the average 14-item PSS score for the participants was computed as 29.89 (range score: 0–52). Our results revealed a comparable stress level when they were compared to a study conducted on medical students in India, where the average PSS scale score was 27.60 [28]. This similarity in both countries indicated that the pandemic had left its effect on the minds of medical students [29]. Another study conducted in Saudi Arabia evaluated stress levels of the university students using the Arabic version of the PSS demonstrated that 86.7% of the participants had moderate- to high-stress levels [30]. Similarly, a Spanish study conducted by Odriozola-González et al. [31] reported moderate to extreme anxiety, depression, and stress scores (21.3%, 34.19%, and 28.14%, respectively) among the university students during the pandemic, which are in line with the stress score of our study. These psychological responses during the time of social distancing might be due to lack of interpersonal communication, the fear of getting infected, and transferring the disease to close family members. Son et al., previously reported increased levels of stress, depressive thoughts, and anxiety in medical students [32]. Our study also reports that most of the participants were moderate to severely stressed due to the pandemic situation. Almost similar stress scores in the above-mentioned studies from different countries in comparison to our study indicate that COVID-19 has affected students around the world similarly. In addition to social distancing, stress can be due to academic, financial, and social difficulties. Coping with the online mode of teaching might also be a challenge for students as they might have faced difficulty in dealing with technology, and faced other problems like absence of stable internet connection, and other online challenges [33]. Our results, when compared with the previously validated studies conducted on healthy populations [31,32,34], showed higher stress, which shows the adverse impact of the pandemic.

In the current study, the mean PSS scores were higher in female participants, with 65% of the female participants showing moderate to severe levels of stress. Another study reported that 73.5% of the females reported moderate to severe stress [35], supporting the current study findings. The female participants of our study were found to be more stressed than males (17% vs. 10%, respectively). A study conducted in South-Western China evaluated stress and anxiety, and the stress scores reported were higher in female quarantined communities during the COVID-19 outbreak when compared with their counterparts [35]. Similarly, another study conducted on undergraduate students in Turkey reported higher stress levels among female students [36]. Earlier studies conducted in Saudi Arabia have reported high-stress scores among different university students, and stress levels were higher among female students [6,17]. The high levels of stress seen in our female participants could possibly be attributed to the fact that males tend to hide their fears due to their conventional gender role [37], which could have led them to report less stress levels in our study. Another plausible reason could be owed to neuroticism (trait of being anxious and emotionally vulnerable), which is found to be more common in females [38], and this could have also resulted in the observation of higher stress levels reported by females in our study. It should also be considered that during the pandemic, mandatory lockdowns were implemented, and females are more at risk of suffering the effects of loneliness on their mental health compared with the males [39], and this could have triggered them to report higher stress levels in our study as well. In addition, in contrast to our study findings, a Chinese study conducted on university students during the COVID-19 outbreak reports no gender-related differences among male and female students regarding stress [40]. In general, medical studies are stressful [41], but a conclusive reason responsible for the different stress levels seen among female and male students could not be determined and requires further investigations.

The COVID-19 has inflicted psychological distress among all population groups [42]. Age-wise, the participants who were less than 20 years old were found to be more stressed, and the PSS score reported in our study decreased linearly with the increasing age of the participants. A previous study has reported that younger people were more vulnerable to depression, stress, and anxiety during the COVID-19 pandemic [43], and our study results are in agreement with that study. Another earlier study reported similar findings and revealed that younger-aged female participants reported more stress levels than all other groups [44]. A probable reason for this finding could be attributed to the fact that younger people worry about their health and academic performance, as shown by an earlier study [45]. On the other hand, older-aged people are better at developing coping strategies to tackle stress [46] and therefore, because of this, they reported lower stress levels in our study.

In the current study, dental assistants/nurses showed the average PSS scores of 27.0, which refers to a moderate stress scale. A Turkish study before the COVID-19 outbreak determined that nursing students face stress levels that could be classified as being above moderate levels [47]. Another study from India indicated moderate levels of PSS scores in nurses with a mean score of 21.88 [48]. Our study also identified moderate levels of stress experienced by the dental assistants/nurses during the pandemic. On the contrary, a study in Norway reported a substantial psychological impact of COVID-19 on dental assistants, causing more stress [49]. The reason for stress seen in this group could be attributed to the fact that dental assistants/nurses have to fulfill their duties even at the time of a pandemic. Lack of personal protective equipment (PPE), discomfort caused by the prolonged usage of PPE, increased workload, along with less experience to deal with the novel virus might have contributed to the stress levels seen in this group [50].

The average PSS score of the faculty in the study indicated the mean score of 28, which indicated that along with the students, the university faculty was equally affected by the pandemic. A study performed in India reported perceived stress to be moderate in dental faculties, which is not in line with our study [51]. However, a study from Norway reported that dental professionals could face increased psychological impacts related to the COVID-19 pandemic [49]. The high-stress score seen in the faculty in our study could be attributed to the fact that dental faculty not only have to be concerned about their own safety, but also for the well-being of their patients, students, and dental assistants as well. They are more vulnerable to infection because of having a close contact with their patients in clinics and while teaching their students during the clinical sessions. A previous study has also reported that dental professionals from all over the world perceive a higher risk of COVID-19 contamination [52]. The lack of knowledge about the controlling of infective virus might have also caused a widespread panic among the faculty in our study. A study conducted in China also reported higher levels of perceived stress in medical staff [53]. It should be noted that psychological stress weakens immunity and makes the person prone to infections [54]; hence, this problem should be tackled as early as possible.

Several countries, including Saudi Arabia, took measures to control the rapidly spreading virus. Citizens were asked to isolate themselves at home and take preventive measures since the advent of the pandemic. Outbreaks like Ebola [55], Severe Acute Respiratory Syndrome (SARS) [56], and MERS [57] have shown some unique concerns related to the mental health of individuals. The situation of lockdown and missing out on major academic tasks (practical sessions and clinical rotations), might have made students more stressed about their future [58], as seen in our study. The effects of COVID-19 are global [59,60] and our study provides a platform for the institute’s policymakers and administrators to provide social assistance to the vulnerable groups.

## 5. Conclusions

It was concluded that the students experienced more stress, followed by the faculty members and dental assistants. In addition, younger participants, females, and senior year students were more stressed than their counterparts. Identifying abnormal stress levels and their timely management and adequate counseling is crucial. It is suggested that in the future, there should be regular checkups of students’ stress levels to evaluate their mental health along with the faculty members and assistants. This could help them to overcome their stresses and address their concerns. Our study results contribute to the literature because our findings highlight that not only students could be stressed, but their teaching faculty members and dental assistants could also be at an increased risk of feeling stressed. We recommend developing the skill of managing distress in a sample of students. We also recommend making a distinction between student risk groups who may not have yet acquired enough skill to manage psychological distress and help them in all possible manners.

## Figures and Tables

**Table 1:** Showing demographics of the participants in our study.

		Frequency	Percentage
Age (years)	26.71 ± 9.26		
Gender	Male	92	35
	Female	173	65
Participants Group	Faculty	41	15
	Student	185	70
	Dental Assistant	39	15
Academic Year Level	2nd Year	35	19
	3rd Year	26	14
	4th Year	33	18
	5th Year	28	15
	6th Year	29	16
	Interns	34	18
Living in Dorm (optional question)	Yes	66	25
	No	154	58

**Table 2:** Showing stress levels of the participants involved in our study. Stress levels are presented as mean (SD).

		Low Stress (0–18)	Moderate Stress (19–37)	High Stress (38–56)	*p*-Value
Age (groups)	Less than 20	2 (4)	35 (75)	10 (21)	0.043 *
	20–30	6 (4)	109 (78)	25 (18)	
	30–40	3 (9)	30 (88)	1 (3)	
	More than 40	3 (12)	22 (88)	0 (0)	
Gender	Male	8 (9)	75 (81)	9 (10)	0.04 *
	Female	7 (4)	137 (79)	29 (17)	
Participants Group	Faculty	6 (15)	33 (80)	2 (5)	0.002 *
	Student	8 (4)	142 (77)	35 (19)	
	Dental Assistant	1 (3)	37 (94)	1 (3)	
Academic Year Level	2nd Year	1 (3)	29 (83)	5 (14)	0.005 *
	3rd Year	0 (0)	21 (81)	5 (19)	
	4th Year	0 (0)	31 (94)	2 (6)	
	5th Year	1 (4)	18 (64)	9 (32)	
	6th Year	1 (3)	17 (59)	11 (38)	
	Interns	5 (15)	27 (79)	2 (6)	

* significant at *p* < 0.05.

**Table 3:** Showing the average PSS scores of the participants involved in our study.

Demographic Variables		Average PSS Score	Standard Deviation	F-Value, *p*-Value
Age (groups)	Less than 20	31.28 ^a^	7.635	6.54, 0.001 *
	20–30	31.04 ^b^	6.941	
	30–40	27.74	5.941	
	More than 40	25.4 ^ab^	6.198	
Participants Group	Faculty	28 ^a^	7.308	7.26, 0.001 *
	Student	31.03 ^a^	7.286	
	Dental Assistant	27.05	4.334	
Academic Year Level	2nd Year	30.00	6.593	4.65, 0.001 *
	3rd Year	31.73 ^a^	5.943	
	4th Year	31.33 ^a^	4.428	
	5th Year	33.04 ^a^	7.928	
	6th Year	34.41 ^a^	7.771	
	Interns	26.74 ^a^	8.28	
Overall PSS Score of Participants		29.89	7.103	

* significant at *p* < 0.05, ^a,b^ same alphabets show significant difference.

**Table 4:** Logistic regression analysis associated with factors possibly related to high stress.

Variables in Equation		OR	Lower 95% CL	Upper 95% CL	Wald X^2^	*p*-Value
Gender	Male	1				
	Female	4.195	1.178	14.943	4.89	0.027 *
Age (groups)	Less than 20	1				
	20–30	0.866	0.166	4.258	0.029	0.865
	30–40	0.363	0.055	2.390	1.111	0.929
	More than 40	0.146	0.018	1.151	3.337	0.068

* significant at *p* < 0.05.
